# Carbolic Acid as a Disinfectant

**Published:** 1897-08

**Authors:** 


### CARBOLIC ACID AS A DISINFECTANT.

Nearly twelve years ago the Committee on Disinfectants of the American Public Health Association, composed of eminent bacteriologists and those interested in public health, made a report which, while it received wide-spread notice, did not receive the attention that its importance demanded. In this report they included the results of a long and careful series of experiments designed to determine the disinfectant or germicidal value of nearly all of the substances which are commonly used for the purpose of destroying germs, and it will be remembered that as a result of their labors we were confirmed in our earlier belief that bichloride of mercury, in proper solution and in proper surroundings, formed at once the most useful and powerful of all disinfectants. Also that, for the disinfection of large masses of material and for cheapness and efficiency, chlorinated lime, when freshly prepared and containing due proportions of chlorine, formed the next best disinfectant, and in many respects was superior to corrosive sublimate itself.

These same studies, moreover, proved beyond doubt, as have many subsequent ones made in other countries, that carbolic acid has a reputation as a germicide far above that which it deserves; that the ordinary solutions of carbolic acid are almost useless as germicides, though they were active as antiseptics; and that it requires very many hours exposure to very strong solutions of carbolic acid to kill pathogenic germs. Notwithstanding these studies, however, and notwithstanding the fact that surgeons have for a number of years ceased to employ carbolic acid for surgical purposes to the extent that they did previously, the laity and some members of the profession seem to regard carbolic acid as the ideal disinfectant, and scatter it about premises and rooms in a manner which, while it assails the nostrils of the individual, does little towards protecting him from infection. Further than this, carbolic acid can be obtained at any drug-store in any quantity upon the request even of a child, and is generally sold without a question being asked as to the purpose for which it is intended. In other words, the laws regarding the sale of poisons do not include carbolic acid in the list of those which shall not be sold without a physician's order. Further, very few of the laity, and some of the profession, seem to be ignorant of the fact that carbolic acid, while it is a poor disinfectant, is, on the other hand, one of the most rapidly acting and lethal poisons known, capable of destroying life within a very few minutes after its ingestion, or of causing death later on by reason of the primary and secondary changes which it produces in the body. In 1892 we called attention to this fact in a leading article in the *Therapeutic Gazette*, our attention being once more called to the matter at that time by the report of four cases of carbolic acid poisoning in *The Chemist and Druggist* for that year.

Our attention has again been called to this matter by an interesting paper by Dr. J. Dixon Mann, professor of Forensic Medicine in Owens College, Manchester, upon "Some Statistics of Carbolic Acid Poisoning," which is published in *The Medical Chronicle* for December, 1896. After mentioning a number of facts which we have already spoken of and pointing out how frequently carbolic acid is handled carelessly and placed in the way of children and ignorant members of the laity, Dr. Mann publishes a table in which he shows that from 1885 to 1895 the total number of deaths from this poison in Great Britain among males was one hundred and seventy-six and among females one hundred and nine; fifty-two of the males were in children under five years of age, and twenty-six of the females were under five years of age. He then goes on to point out that carbolic acid stands fourth among the poisons by which death was accidentally or negligently caused during the ten years ending 1894, and if chloroform and lead, the first of which has produced accidental death in a large number of cases and the second of which produces poisoning in the arts, are excluded, carbolic acid takes the second place among the poisons which have caused accidental death from personal carelessness or ignorance.

Again, Dr. Mann points out that in the ten years which we have mentioned one-fourth of the women who committed suicide by poison did so with carbolic acid, and it ranks second as a suicidal poison for males; while if the two sexes are taken together carbolic acid stands first of all poisons used for the purpose of committing suicide. It is also interesting to note that the number of people who commit suicide by means of carbolic acid has rapidly progressed each year: thus in 1891 there were 63; in 1892, 73; in 1893, 117; and in 1894, 167. He also mentions two cases in which carbolic acid was used for the purpose of committing murder, the victims being children; and two cases of manslaughter also occured.

Finally, he sums up the facts that during the ten years we have named 917 deaths were due to carbolic acid, an average of nearly 100 deaths; and that in 1894 the number of deaths from this cause reached the appalling total of 202.

Dr. Mann urges, therefore, what we have previously urged, that a restriction should be put upon the sale of this violent poison; and we doubt not that an examination of statistics in this country would give conclusions nearly identical with those obtained by Dr. Mann from a study of the statistics in the United Kingdom of Great Britain.—*Editorial in Therapeutic Gazette.*

